# Antifungal amphiphilic aminoglycoside K20: bioactivities and mechanism of action

**DOI:** 10.3389/fmicb.2014.00671

**Published:** 2014-12-05

**Authors:** Sanjib K. Shrestha, Cheng-Wei T. Chang, Nicole Meissner, John Oblad, Jaya P. Shrestha, Kevin N. Sorensen, Michelle M. Grilley, Jon Y. Takemoto

**Affiliations:** ^1^Department of Biology, Utah State UniversityLogan, UT, USA; ^2^Synthetic Bioproducts Center, Utah State UniversityNorth Logan, UT, USA; ^3^Department of Chemistry and Biochemistry, Utah State UniversityLogan, UT, USA; ^4^Department of Immunology and Infectious Diseases, Montana State UniversityBozeman, MT, USA; ^5^Department of Biology, Snow CollegeEphraim, UT, USA

**Keywords:** antifungal, amphiphilic aminoglycoside, K20, *Cryptococcus neoformans*, kanamycin

## Abstract

K20 is a novel amphiphilic antifungal aminoglycoside that is synthetically derived from the antibiotic kanamycin A. Reported here are investigations of K20′s antimicrobial activities, cytotoxicity, and fungicidal mechanism of action. *In vitro* growth inhibitory activities against a variety of human and plant pathogenic yeasts, filamentous fungi, and bacteria were determined using microbroth dilution assays and time-kill curve analyses, and hemolytic and animal cell cytotoxic activities were determined. Effects on *Cryptococcus neoformans* H-99 infectivity were determined with a preventive murine lung infection model. The antifungal mechanism of action was studied using intact fungal cells, yeast lipid mutants, and small unilamellar lipid vesicles. K20 exhibited broad-spectrum *in vitro* antifungal activities but not antibacterial activities. Pulmonary, single dose-administration of K20 reduced *C. neoformans* lung infection rates 4-fold compared to controls. Hemolysis and half-maximal cytotoxicities of mammalian cells occurred at concentrations that were 10 to 32-fold higher than fungicidal MICs. With fluorescein isothiocyanate (FITC), 20–25 mg/L K20 caused staining of >95% of *C. neoformans* and *Fusarium graminearum* cells and at 31.3 mg/L caused rapid leakage (30–80% in 15 min) of calcein from preloaded small unilamellar lipid vesicles. K20 appears to be a broad-spectrum fungicide, capable of reducing the infectivity of *C. neoformans*, and exhibits low hemolytic activity and mammalian cell toxicity. It perturbs the plasma membrane by mechanisms that are lipid modulated. K20 is a novel amphiphilic aminoglycoside amenable to scalable production and a potential lead antifungal for therapeutic and crop protection applications.

## Introduction

Fungal diseases are major threats to human health and food security (Strange and Scott, 2005; Fisher et al., 2012). Invasive human fungal infections such as cryptococcal meningitis caused by *Cryptococcus neoformans* have increased due to the rising number of immunocompromised individuals (Park et al., 2009; Shirley and Baddley, 2009). Fungal crop diseases such as wheat head blight or scab (caused by *Fusarium graminearum*) and stem rust (caused by *Puccinia graminis*) create large economic losses and threats to the world's food supplies (Strange and Scott, 2005). Conventional antifungals such as amphotericin B, and azoles are still used to treat invasive fungal infections (Jarvis and Harrison, 2008) and fungicidal triazoles and strobulins continue to be used in massive quantities for wheat and other major crops (Fisher et al., 2012; Strange and Scott, 2005). Their effectiveness however grows increasingly limited by fungal resistance, host side effects, and ecosystem disturbances (Fisher et al., 2012; Strange and Scott, 2005). A consequence is a growing need to develop novel antifungals that are safe and effective.

**Figure 1:**
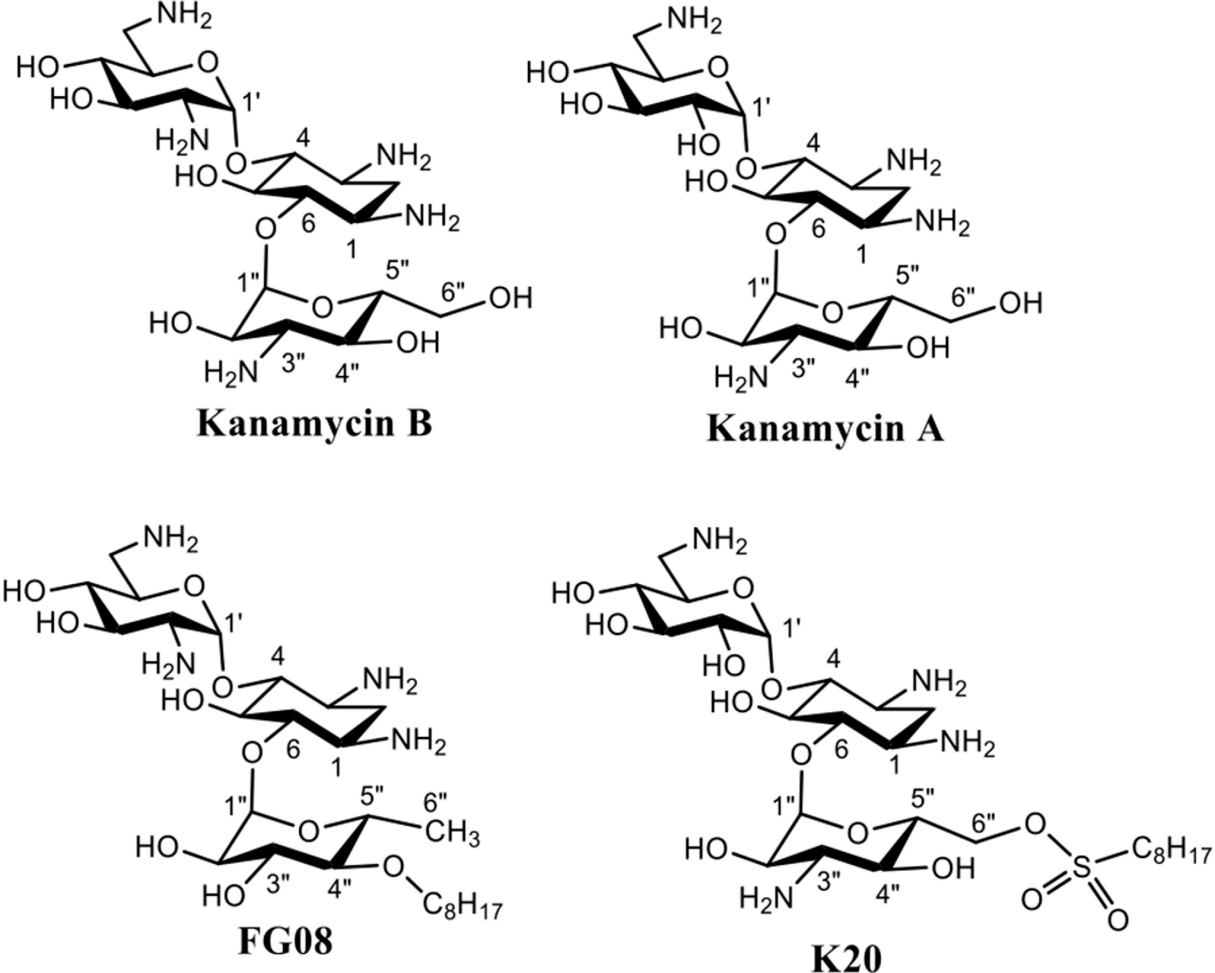
**Structures of aminoglycosides FG08, kanamycin A, and K20**.

Aminoglycosides are compounds having two or more amino sugars bound to an aminoacyclitol ring via glycosidic bonds. Many are used therapeutically against bacterial infections of humans and animals (Jarvis and Harrison, 2008). Among them, kanamycin A, produced by the soil microbe *Streptomyces kanamyceticus*, is one of the most successful (Umezawa et al., 1957; Begg and Barclay, 1995; Vakulenko and Mobashery, 2003). Kanamycin A is structurally based on neamine rings I and II with an attached ring III of *O*-6-linked kanosamine (Figure 1). Most bind to the prokaryotic 16S rRNA in the decoding region A site, leading to the formation of defective cell proteins. Despite being mainly antibacterial, certain classical aminoglycosides are also found to inhibit crop pathogenic fungal-like heterokonts (Lee et al., 2005) and certain structurally unusual ones inhibit yeasts and protozoans (Wilhelm et al., 1978). Previously, we reported on a novel aminoglycoside analog of kanamycin B, FG08, with broad-spectrum antifungal properties that did not inhibit tested bacterial and mammalian cells (Figure 1) (Chang et al., 2010). FG08 differs from kanamycin B by substitution of a C8 alkyl chain at the *O*-4″position of ring III to impart amphiphilic properties (Figure 1) (Chang et al., 2010). However, as a lead antifungal agent, FG08 is limited. Incorporation of the C8 alkyl chain at the kanamycin B *O*-4″ position is difficult and the product yield is low. These shortcomings prompted the search for similar amphiphilic aminoglycosides using alternative synthetic approaches (Chang and Takemoto, 2014). From this effort, a novel and scalable aminoglycoside, K20, derived from kanamycin A was discovered that structurally resembled FG08 and that also possessed antifungal activity (Chang and Takemoto, 2012, 2014).

In the current study, K20′s antifungal activities are more thoroughly examined, and its animal cell cytotoxicity and hemolytic capabilities were determined. K20′s antifungal mechanism of action was determined using intact fungal cells and model lipid bilayer membranes. Like FG08, K20 exhibited growth inhibitory activities against a broad range of fungal species, but not against bacteria, and it was not hemolytic or cytotoxic at concentrations that inhibit fungi. K20′s primary mechanism of action is shown to involve perturbation of plasma membrane permeability. Finally, in proof of concept experiments, K20 was observed to reduce the infectivity of *C. neoformans* in a preventive murine lung infection model,

## Materials and methods

### K20 and other antimicrobials

K20 was synthesized from kanamycin A (Chang and Takemoto, 2014). Briefly, tetra-di-*tert*-butyl carbonate (Boc)-protected kanamycin A was stirred overnight with octanesulfonyl chloride in anhydrous pyridine at 0°C. The mixture was then stirred at room temperature for 6 days, heated and incubated at 40°C for 1 day, and then concentrated to an oily crude product. Water (500 mL) was added to the residue material, and the mixture was stirred for 1 day. The suspension was extracted with ethyl acetate in a separatory funnel, washed twice with 1.0 N HCl and once with water. The wash sequence was repeated 3 to 4 times, and the final organic layer was filtered and evaporated. The residue was treated with trifluoroacetic acid/dichloromethane (1:4) and stirred overnight. The solvents were removed, water added and the material evaporated to remove residual acid. The crude product was dissolved in water and washed repeatedly with ethyl acetate until the aqueous fraction was clear. The aqueous solution was concentrated and passed through a column of Dowex1X-8 (Cl-form). K20 in highly pure form was recovered (overall yield of 40%) after evaporation and stored as a solid at 5**°**C. K20 was characterized by ^1^H NMR and ^13^C NMR (using a Joel 300 MHz NMR spectrometer) and mass spectrometry [using a Waters GCT (2008) High resolution mass spectrometer at the High Resolution Mass Spectrometry Facility, University of California, Riverside, USA]. Correlation Spectroscopy (COSY) and edited Heteronuclear Single Quantum Correlation (HSQC) were used for H-H and H-C correlation, respectively (see Supplementary Material). For bioactivity tests and mechanism of action studies, a 10 mg/mL stock solution was prepared in twice distilled water and stored at 5°C. FG08 was synthesized as previously described (Chang et al., 2010), and kanamycin A was purchased (Changzhou Zhongtian Chemical Co. LTD., Changzhou, PRC).

### Organisms and culture conditions

*Fusarium graminearum* strain B4-5A was obtained from the Small Grain Pathology Program, University of Minnesota, Minneapolis MN, USA. *E. coli* TG1, *S. aureus* ATCC6538, *M. luteus* ATCC10240, *C.albicans* ATCC10231(azole-resistant), *C. albicans* ATCC64124 (azole–resistant), and *C. albicans* ATCC MYA-2876 (azole sensitive) were obtained from the American Type Culture Collection (Manassas, VA, USA). *Saccharomyces cerevisiae* strains W303C (*MATa ade2 his3 leu2 trp1 ura3*) and isogenic sphingolipid biosynthesis mutant strains W303-Δ*syr2* (*MATα ade2 his3 leu2 trp1 ura3 syr2 (sur2)::URA3*), W303-Δ*elo3 (MATα ade2 his3 leu2 trp1 ura3 elo2::HIS3)*, and W303-Δ*syr4(ipt1)(MATα ade2 his3 leu2 trp1 ura3 syr4 (ipt1)::URA3)* were previously described (Stock et al., 2000). Phenotypically, these mutants lack sensitivity to the antifungal syringomycin E—a membrane lipidic pore forming cyclic lipodespsipeptide (Stock et al., 2000). *C. neoformans* H99 was obtained from Dr. J. Perfect (Duke University Medical Center, Durham, NC, USA). C. *neoformans* 94-2586, *C. neoformans* 90-26, *C. tropicalis* 95-41,*C. albicans* 94-2181, *C. albicans* B-311, *C. rugosa* 95-967, *C. pseudotropicalis* YOGI, and *C. lusitaniae* 95-767 were obtained from the laboratory culture collection of Dr. Kevin Sorensen (Snow College, Ephraim, Utah, USA). *Aspergillus flavus*, and *F. oxysporum* were obtained from Dr. Bradley Kropp (Utah State University, Logan, UT, USA) and *A. niger* and *Botrytis alcada* were obtained from Dr. Claudia Nischwitz (Utah State University, Logan, UT, USA). Filamentous fungi and yeast strains were maintained on potato dextrose agar (PDA) and cultivated at 28°C in potato dextrose broth (PDB) or at 35°C with RPMI 1640 (with L-glutamine, without sodium bicarbonate (Sigma-Aldrich Chemical Co., St. Louis, MO, USA) buffered to pH 7.0 with 0.165 M morpholinepropanesulfonic acid (MOPS). Bacterial strains were grown at 37°C for 24 h on Luria-Bertani (LB) medium (Sambrook et al., 1989) except for *S. aureus* ATCC6538 which was grown on Mueller-Hinton medium (Difco, BD, Franklin Lakes, NJ, USA).

### Fungal growth inhibition assays

Minimal inhibitory concentration (MIC) and minimal fungicidal concentration (MFC) values of K20 against yeast strains were determined using microbroth dilution assays in 96-well uncoated polystyrene microtiter plates (Corning Costar, Corning, NY, USA) as described in the M27-A3 reference methods of the Clinical and Laboratory Standards Institute (CLSI) (formerly the National Committee for Clinical Laboratory Standards) (NCCLS, 2002) with minor modifications. Modifications included growing yeast cell inocula in RPMI 1640 medium for 48 h at 35°C and suspending fresh-grown inocula to a concentration of 5 × 10^4^ cells/mL (determined by hemocytometer cell counting) in fresh RPMI 1640 for the assays. All yeast cell suspensions (100 μL) containing 0.48 to 250 mg/L of serial diluted K20 except for *C. neoformans* (with 0.25–128 mg/L of K20) were added to the wells of a 96-well microtiter plate and incubated for 48 h at 35°C. Controls were no yeast cells and no K20 added to separate wells. MFC values were determined as the occurrence of fewer than 3 colonies after plating 5 μL of the cleared microtiter plate wells from MIC tests on Sabouraud's dextrose agar medium (Difco, BD, Franklin Lakes, NJ, USA). Each test was performed in triplicate. For *in vitro* antifungal activities against *F. graminearum* B4-5A, *F. oxysporum*, *A. flavus*, *A. niger*, and *Botrytis alcada*, spores were prepared as described previously (Lay et al., 2003). Spores were isolated from sporulating cultures growing in PDB medium by filtration through sterile glass wool. Microbroth dilution assays for determination of MICs were conducted using the M38-A2 protocols of the CLSI (NCCLS, 2008) with minor modification. Serial dilutions of K20 were made in uncoated polystyrene 96-well plates in the range of 0.48–250 mg/L using RPMI 1640 medium and spore suspensions were added to make a final concentration of 5 × 10^5^ CFU/mL. The plates were incubated at 35°C for 72 h except for tests with *F. graminearum* B4-5A which were incubated for 48 h. MIC values were determined as the lowest concentration of compounds showing optically clear solutions by visual inspection of the plate wells (NCCLS, 2002, 2008). Each test was performed in triplicate. Disk diffusion assays of yeast strains were performed as previously described (Chang et al., 2010). Cell suspensions (0.5 mL)were spread–plated onto potato-dextrose agar (PDA) medium and air-dried for 5 min. Eight microliter aliquots of K20 (1–10 mg/mL in water) were applied to 0.6 cm diameter paper disks placed on the agar surfaces, and the plates were incubated for 24–48 h at 28°C. These amounts of K20 provided visible and measurable zones of growth inhibition around the disks as previously determined for FG08 (Chang et al., 2010).

### Bacterial growth inhibition assays

The *in vitro* effects of K20 on the growth of bacterial species *E. coli* TG*1, M. luteus* ATCC10240 and *S. aureus* ATCC6538 were assayed in 96-well uncoated polystyrene microtiter plates and MICs were determined using CLSI protocols with modification (NCCLS, 1993). Cells were grown overnight in Luria-Bertani medium and diluted to a concentration of 1 × 10^4^ CFU/mL. Ten microliter of the diluted overnight culture were then added to 190 μL of Luria–Bertani medium containing K20 at concentrations ranging between 0.48 and 250 mg/L. Controls were bacterial cells only and no K20 added to separate wells. The plates were incubated at 37°C without shaking for 24 h before determination of MICs. Experiments were performed in triplicate.

### Antifungal carryover and time-kill curve analyses

Antifungal carryover was determined as described by Klepser et al. (2000). *C. neoformans* H99 cell suspensions were prepared in sterile water to yield 1 × 10^5^ CFU/mL. One hundred microliter of each suspension was added to 900 μL of sterile water (control) or to sterile water containing K20 at concentrations of 2, 4, and 8 mg/L, equal to 0.5, 1, and 2 times the MIC, respectively. Immediately after addition of fungal suspension, 100 μL of suspension was removed and spread-plated on PDA for colony count determination. Antifungal carryover was indicated when a reduction in colony counts of >25% compared to controls was observed. Time-kill curves were generated as described (Klepser et al., 2000) with modifications. Colonies from 24 to 48 h cultures were suspended in 9 mL sterile water and adjusted to 1 × 10^8^ CFU/mL. One milliliter of the adjusted fungal suspension was then added to 1 L of either PDB growth medium alone (control) or a solution of PDB and K20 at concentrations of 2, 4 or 8 mg/L. Fifty milliliter aliquots of culture suspensions in 125-mL capacity Erlenmeyer flasks were incubated in a water bath shaker (Model G76, New Brunswick Scientific, NJ, USA) with agitation at 35°C. At 0, 4, 9, 24, and 48 h, 100 μL aliquots were removed from each solution and serially diluted 10-fold in sterile water. One hundred microliter volumes of each dilution were spread on agar surfaces of potato dextrose agar [PDB containing agar (2%, wt/vol)] plates to allow growth. Colony counts were determined after incubation for 48 h. The experiment was performed in triplicate. The lower limit for accurate and reproducible quantification was 50 CFU/mL (Klepser et al., 2000).

### Hemolytic activity

Hemolytic activity was determined using previously described methods (Dartois et al., 2005) with modification. Sheep erythrocytes were obtained by centrifuging sheep whole blood at 1000× *g*, washing four times with phosphate-buffered saline (PBS), and resuspending in PBS to a final concentration of 10^8^ erythrocytes/mL. The erythrocyte suspension (80 μL) was added to wells of a 96-well polystyrene microtiter plate containing 20 μL of serially diluted K20 (1.0–0.015.1 g/L) in water. The plate was incubated at 37°C for 60 min. Wells with added deionized water and Triton X-100 (1% v/v) served as negative (blank) and positive controls, respectively. The A_490_ values of each well were measured using a BioTek Synergy 4 microplate reader (BioTek Instruments Inc., Winooski, VT, USA). Percent hemolysis was calculated using the following equation: % hemolysis = [(A_490_ of sample) − (A_490_ of blank)] × 100/(A_490_ of positive control). Fifty percent hemolysis (HC_50_) values were calculated as K20 concentrations that lyse 50% of the erythrocytes.

### *In vitro* cytotoxicity assays

Cytotoxicity assays were performed as previously described for FG08 (Shrestha et al., 2013). The C8161.9 melanoma cell line was a gift from Dr. Danny R. Welch, University of Kansas, Lawrence, KS (USA). Fibroblast cell line NIH3T3 (ATCC® CRL-1658™) was obtained from the American Type Culture Collection (Manassas, VA, USA.)

C8161.9 cells were grown in DMEM/Ham's F12 (1:1) containing 10% fetal bovine serum (FBS). NIH3T3 cells were grown in DMEM (high glucose) medium containing 10% FBS in Corning Cell Bind flasks. The confluent cells were then trypsinized with 0.25%, w/v trypsin and resuspended in fresh medium (DMEM). The cells were transferred into 96-well uncoated polystyrene microtiter plates at a density of 2 × 10^5^ cells/mL. K20 was added at final concentrations of 10, 20, 50, 100, and 250 mg/L or an equivalent volume of sterile double distilled water (negative control). The cells were incubated for 24 h at 37°C with 5% CO_2_ in a humidified incubator. To evaluate cytotoxicity, each well was treated with 10 μL of 3-(4,5-dimethylthiazol-2-yl)-2,5-diphenyltetrazolium bromide (MTT) (Sigma-Aldrich, St. Louis, MO USA) for 4 h. In living cells, mitochondrial reductases convert the MTT tetrazolium to formazan, which precipitates. Formazan was dissolved adding 10% (wt/vol) NaDodSO4 in 0.01 M HCl and quantified at A_570_ using a BioTek Synergy4 microplate reader (BioTek Instruments Inc., Winooski, VT, USA). Triton X-100® (1%, vol/vol) gave complete loss of cell viability and was used as the positive control. The ratios of A_570_ values for K20 treated cells to the A_570_ values for the untreated cells were used to calculate % cell survival. Standard deviations were determined from data sets of three separate experiments.

### Membrane permeabilization

*C. neoformans* H99 (5 × 10^5^ CFU/mL) or *F. graminearum* (5 × 10^5^ conidia/mL) were grown for 18 h in PDB with continuous agitation. Aliquots (500 μL) were taken and centrifuged for 2 min at 10,000× *g*. The fungal pellet was suspended in 10 mM HEPES, pH 7.4, centrifuged again, and suspended in 500 μL distilled water (Chang et al., 2010). *C. neoformans* H99 cells were exposed to 4, 8, and 25 mg/L K20 and *F. graminearum* B4-5A hyphae to 7.8, 15.6, and 32 mg/L K20 for 1 h at 28°C with continuous agitation. The K20 treated fungi were assessed 10 min after addition of fluorescein isothiocyanate (FITC) (10 mg/mL stock in acetone) (Sigma-Aldrich Chemical Co., St. Louis, MO, USA) to 6 mg/L as previously described (Shrestha et al., 2013) with slight modification. Negative (water) and positive (Triton X-100® 1%, vol/vol) treatment controls were also prepared. Glass slides were prepared with 10 μL of each mixture and observed in dark-field and fluorescence (using an Olympus MWIB filter, excitation, and emission wavelength 488–512 nm) modes with an Olympus IX81 fluorescence microscope (Olympus, Center Valley, PA, USA). Dye uptake of *C. neoformans* H99 cells was quantitated as previously described (Shrestha et al., 2013) and of *F. graminearum* B4-5A by qualitative estimates from visual inspection. Data were obtained from at least three independent experiments.

### Calcein release from small unilamellar vesicles (SUVs)

Lipids (from Sigma-Aldrich Chemical Co., St. Louis, MO USA) were phosphatidylcholine from *Glycine max* (PC), L-α-phosphatidylethanolamine from *E. coli* (PE), L-α- phosphatidylinositol (Na salt) from *G. max* (PI), and ergosterol. Model lipid bilayer membrane SUVs were prepared by dissolving mixtures of lipids in chloroform/methanol (2:1, vol/vol). The mixtures were PC, PE, PI, and ergosterol (5:4:1:2 ratios by wt) and PC and ergosterol (10:1 ratio by wt) to mimic the lipid compositions of fungal plasma membranes (Makovitzki et al., 2006; Lee et al., 2009). The organic solvents were evaporated with nitrogen gas and the lipid mixtures dried under vacuum. The dried lipid films were rehydrated in HEPES buffer (10 mM HEPES, 150 mM NaCl, pH 7.4) and sonicated to generate SUVs with lipid concentrations at 10 mg/mL. Lipid films were prepared as described above and were suspended in 10 mM HEPES, 150 mM NaCl, pH 7.4, and 60 mM calcein (self-quenching concentration) (Makovitzki et al., 2006). Liposome suspensions were sonicated for 2 min using a sonicator (Sonicator™ Heat System, W-220F, Ultrasonics, NY, USA). The free calcein was removed by gel filtration through a Sephadex G-50 column. K20 at concentrations of 31.3 [at or near the MICs for most fungi tested (Table 1)], 62.2, and 125 mg/L (2- and 4-fold higher, respectively, than the initial concentration) was added to the calcein-loaded SUV suspensions (lipid concentration of 6 to 10 μM), and calcein leakage was followed by measuring fluorescence using a BioTek Synergy HT microplate reader at an excitation wavelength of 488 nm and emission wavelength of 520 nm. Complete (100%) dye release was obtained by addition of Triton X-100**®** (1%, vol/vol). The dye-leakage percentage was calculated as follows: % dye leakage = 100×(*F*-*F*_0_)/(*F*_t_-*F*_0_), where *F* represents the fluorescence intensity 2 min after K20 addition, and *F*_0_ and *F*_t_ represent the fluorescence intensity without K20 and with Triton X-100**®**(1%, vol/vol), respectively (Zhang et al., 2001).

**Table 1:** **Minimal inhibitory concentrations of K20 and kanamycinA against bacteria and fungi**.

**Organism**	**MIC (mg/L)^a^**			
	**K20**	**Kanamycin**	**ITC**	**FLC**
**YEASTS**				
*C. neoformans* H99	3.9–7.8	>125^b^	1.56	1.56
*C. neoformans* 94-2586	3.9–7.8	>125^b^	0.06	1.56
*C.neoformans* 90-26	3.9–7.8	>250	0.37	>0.195
*C. pseudotropicalis* YOGI	15.6	>250	0.125–0.8	nd^c^
*C. lusitaniae* 95-767	>7.8	>250	0.2	1.56
*C. rugosa* 95-967	15.6	>250	0.12	>0.78
*C. tropicalis* 95-41	15.6	>250^b^	>25	>25
*C. albicans* 10231	15.6	>250^b^	0.75	25
*C. albicans* 64124(R)^d^	31.3	>500^b^	>64	>200
*C. albicans* MYA 2876 (S)^e^	15.6	>250	>2	1.56
*C. albicans* B-311	>7.8	>250	16–32	>25
*C. albicans* 94-2181	>7.8	>250	>8–16	>12.5
*C. parapsilosis* (R)^d^	15.6–31.3	>250	0.5	>16
*C. parapsilosis* (S)^e^	15.6	>250	0.015	0.12
**FILAMENTOUS FUNGI**				
*F. graminearum* B-4-5A	7.8	>125^b^	nd	nd
*F. oxysporum*	31.3	>250^b^	nd	nd
*A. flavus*	300	>250	0.125	nd
*A. niger*	>150	>250	nd	nd
*B. alcada*	15.6		nd
**BACTERIA**				
*E. coli* TG1	125–250	1.95^b^		
*S. aureus* ATCC 25923	250	<0.98^b^		
*M. luteus* ATCC10240	62.5	1.95^b^		

a *Microbroth dilution assays were performed at least twice, and each in triplicate*.

b *Determined with kanamycin A; all others were determined with kanamycin B*.

c *not determined*.

d *(R) Resistant*.

e *(S) Sensitive*.

### Cryptococcosis preventive murine lung infection model

*In vivo* efficacy of K20 treatment was evaluated in a proof of concept study using preventive murine lung infection model as previously described (Searles et al., 2013). For this study RAG^−/−^ mice lacking both T and B cells due to a defect in the recombination antigen gene were obtained from Jackson Laboratories (Bar Harbour, ME, USA) and maintained at the Montana State University Animal Resource Center (Bozeman, MT, USA). The studies conformed to NIH guidelines and were approved by the Montana State University IACUC and biosafety committee (approval number: (2014-17 and 027-2013). Three treatment groups each consisting of five RAG^−/−^ mice were compared. Group A received one dose of 100 μL of 200 mg/L K20 in PBS (10 mM phosphate, 2.7 mM KCl, and 137 mM NaCl, pH 7), group B received 100 μL of 200 mg/L K20 mixed with *C. neoformans* H99 cells (5 × 10^3^ cells/mL), and group C received cells mixed in PBS only by intratracheal instillation. K20 doses averaged 0.824 mg/kg body weight. Mice were monitored for signs of distress and their weights recorded daily over the course of infection. Weight loss or gain was plotted as percent of weight change. Mice were euthanized if weight loss exceeded 25%. At day 15 post-infection, lungs were removed, suspended in 5 mL PBS and homogenized by extrusion through a 100 μm mesh steel screen. Lung cryptococcal burden was assessed by plating 100 μL of the homogenized suspension onto yeast extract-peptone-dextrose agar plates (26) at 1:10, 1:100, and 1:1000 dilutions in PBS, incubated for 3 days, and colonies counted. For microscopic examination, homogenates were suspended in 10 mL of PBS and a 1:20 dilution of the homogenate was spun onto glass slides using a Cytospin 4 centrifuge (ThermoFisher, NJ, USA). Slides were fixed in methanol for 3 min followed by Diff-Quik™ (Siemens Healthcare Diagnostics Inc. Newark, DE USA) staining for 3 min each in solution 1 and 2. Stained yeast cells were visualized with a Nikon 80i Eclipse upright microscope as large purple colored cells surrounded by opaque halos. The experiments were performed twice. Data were statistically analyzed and *P*-values determined by one-way (lung burden experiments) and Two-Way (weight change experiments) ANOVA methods using GraphPad Prism software (La Jolla, CA, USA).

## Results

### *In vitro* antifungal and antibacterial activities

**Figure 2:**
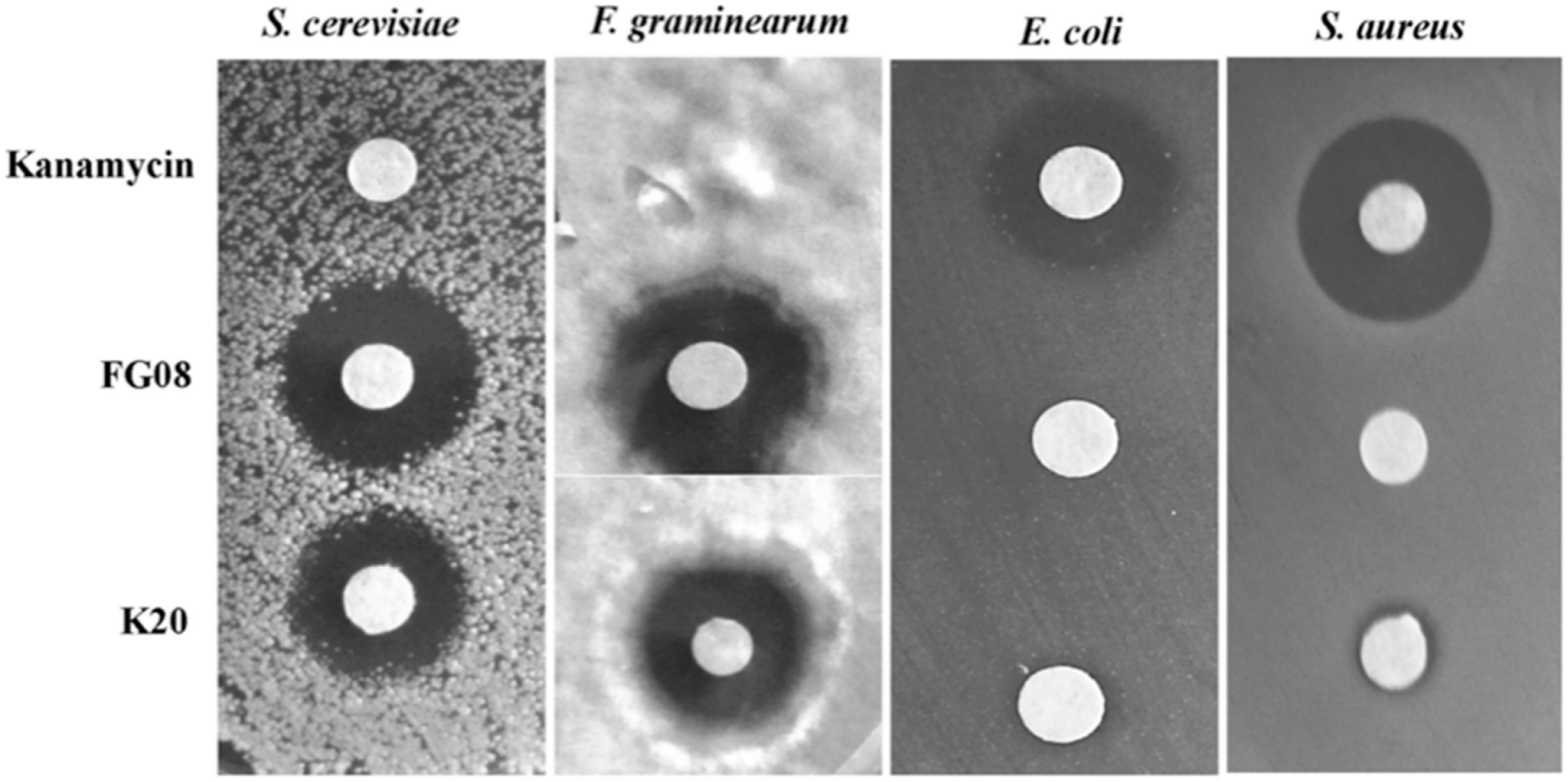
**Antimicrobial activities of K20, FG08, and kanamycin A**. Disk agar diffusion assays show that K20 and FG08 are antifungal, but not antibacterial. Kanamycin A is antibacterial, but not antifungal. Ten μL aliquots of K20, FG08, and kanamycin A solutions were applied to paper disks (0.6 cm diameter) on surfaces of PDA and LB agar at concentrations of 10 and 5 mg/mL, respectively, and with spread-plated fungal (*S. cerevisiae* strain W303C and *F. graminearum* B4-5A) and bacterial (*E. coli* TG1and *S. aureus* ATCC 6538) cultures, respectively.

K20 generally displayed antifungal activities against yeasts (e.g., *S. cerevisiae* strain W303C) and filamentous fungi (e.g., *F. graminearum* B4-5A), but no or little activity against either Gram negative (e.g., *E. coli* TG1) or Gram-positive (e.g., *S. aureus* ATCC 6538) bacteria (Figure 2). In microbroth dilution assays with RPMI 1640 medium, K20 inhibited the growth of most fungi tested (Table 1). MICs ranged from 4 to 31.3 mg/L for yeasts and 7.8–300 mg/L for filamentous fungi (Table 1). K20 MICs with yeasts were uniformly higher than MICs achieved with itraconanzole and fluconazole except with azole resistant strains, *C. albicans* strains 64124 and B-311 and *C. tropicalis* 95-41. MFC values determined for *C. albicans* strains MYA 2876 and 64124 were equal to or 2-fold higher than the corresponding MIC values (data not shown). Among the yeasts tested, *C. neoformans* strains were consistently the most susceptible to K20. Among filamentous fungi tested, *F. graminearum* B4-5A was the most susceptible to K20; *A. flavus* and *A. niger* were the least susceptible. Bacterial species *E. coli* TG*1, M. luteus* ATCC10240 and *S. aureus* ATCC6538 growing in LB medium were less susceptible to K20. The antibacterial MICs were 65 to 125-fold higher than shown by kanamycin A (Table 1).

### Antifungal carryover and time-kill analyses

**Figure 3:**
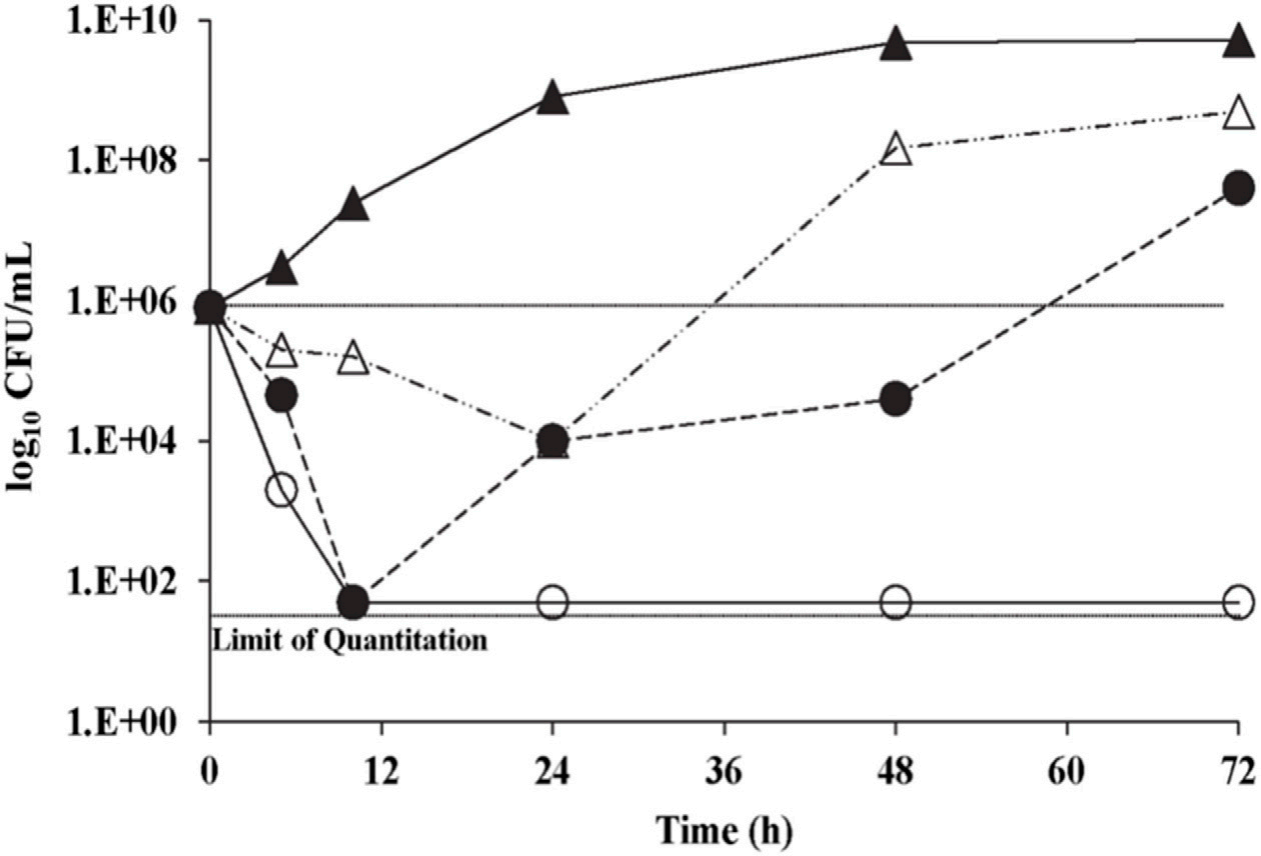
**Time kill curves for *C. neoformans* H99 exposed to K20**. Cultures were exposed to K20 at 2 mg/L (open triangles), 4 mg/L (filled circles), and 8 mg/L (open circles) or to no K20 (filled triangles).

With *C. neoformans* H99, no antifungal carryover was observed in the procedures used at 0.5, 1, and 2× the K20 MIC. The time kill curves for K20 and *C. neoformans* H99, showed that the MIC level of K20 (4 mg/L) reduced the CFU/mL by ≥2 log_10_ units (Figure 3). However, a 2× MIC level (8 mg/L) of K20 was required to achieve a fungicidal effect (100% killing). At 4 mg/L (1× MIC), K20 exhibited a fungistatic effect after 10 h incubation.

### Sheep erythrocyte hemolysis

**Figure 4:**
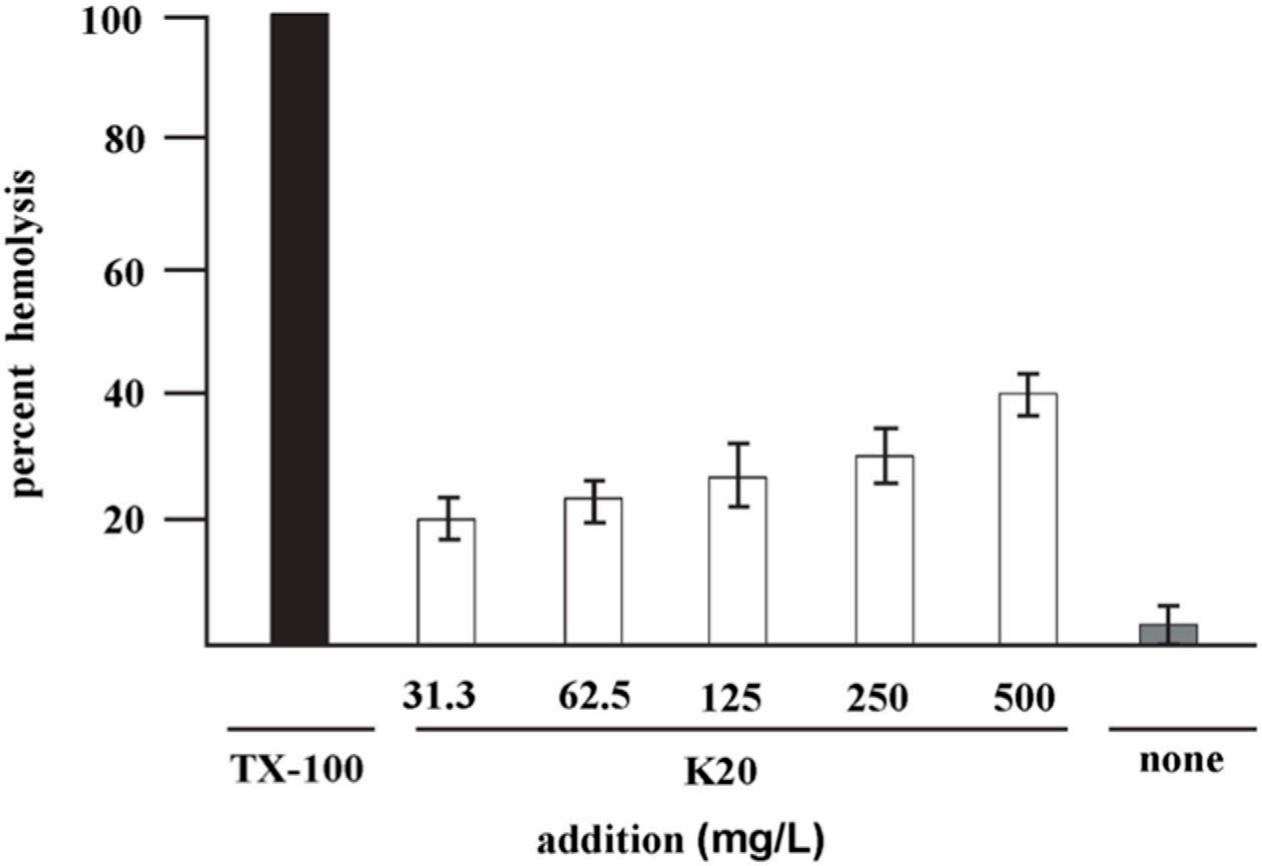
**Hemolysis of sheep erythrocytes with various concentrations of K20 after 1 h exposure at 37°C (white bars)**. Controls were exposure to Triton X-100^®^ (1%, vol/vol) giving 100% hemolysis (black bar) and no exposure to K20 (gray bar). The HC50 value is >500 mg/L. Standard deviation was used as the statistical parameter.

K20 lysed <40% of sheep erythrocytes at 500 mg/L (Figure 4) a concentration that is >50-fold higher than the antifungal MIC against *C. neoformans* H99. The HC_50_ value for K20 was >500 mg/L. Kanamycin A did not show hemolytic activity against sheep erythrocytes (data not shown).

### Animal cell cytotoxicity

**Figure 5:**
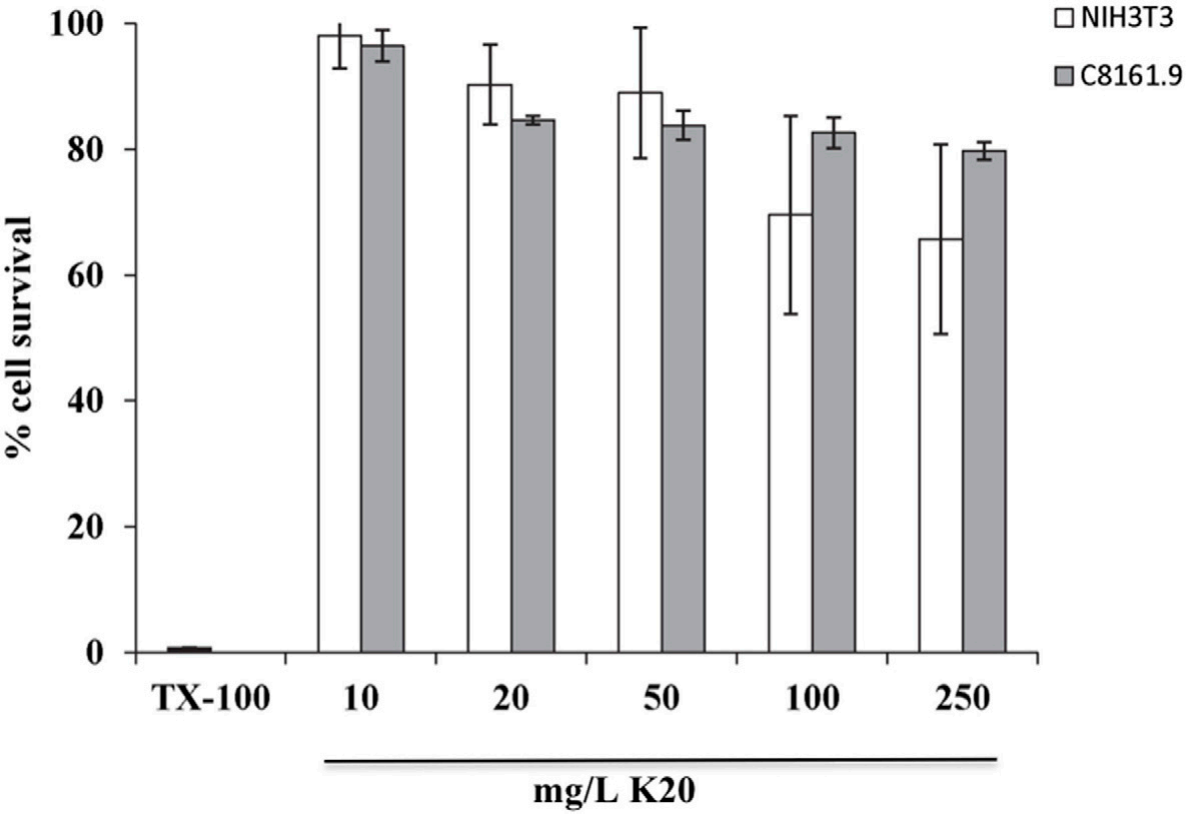
**Toxicities of NIH3T3 mouse fibroblast cells (white bars) and C8161.9 melanoma cells (gray bars) with 24 h exposure to K20 at various concentrations**. Positive control (0% cell survival) was provided by treatment with Triton X100^®^ (1%, vol/vol) (black bar).

K20 showed no or low toxicity against C8161.9 and NIH3T3 cells at concentrations up to 250 mg/L (Figure 5). The 50% inhibitory concentrations (IC_50_) of K20 for both C8161.9 and NIH3T3 cells were > 500 mg/L (Figure 5), and at least 31-fold higher than the antifungal MIC against *C. neoformans* H99 (Table 1).

### Fluorescent dye uptake

**Figure 6:**
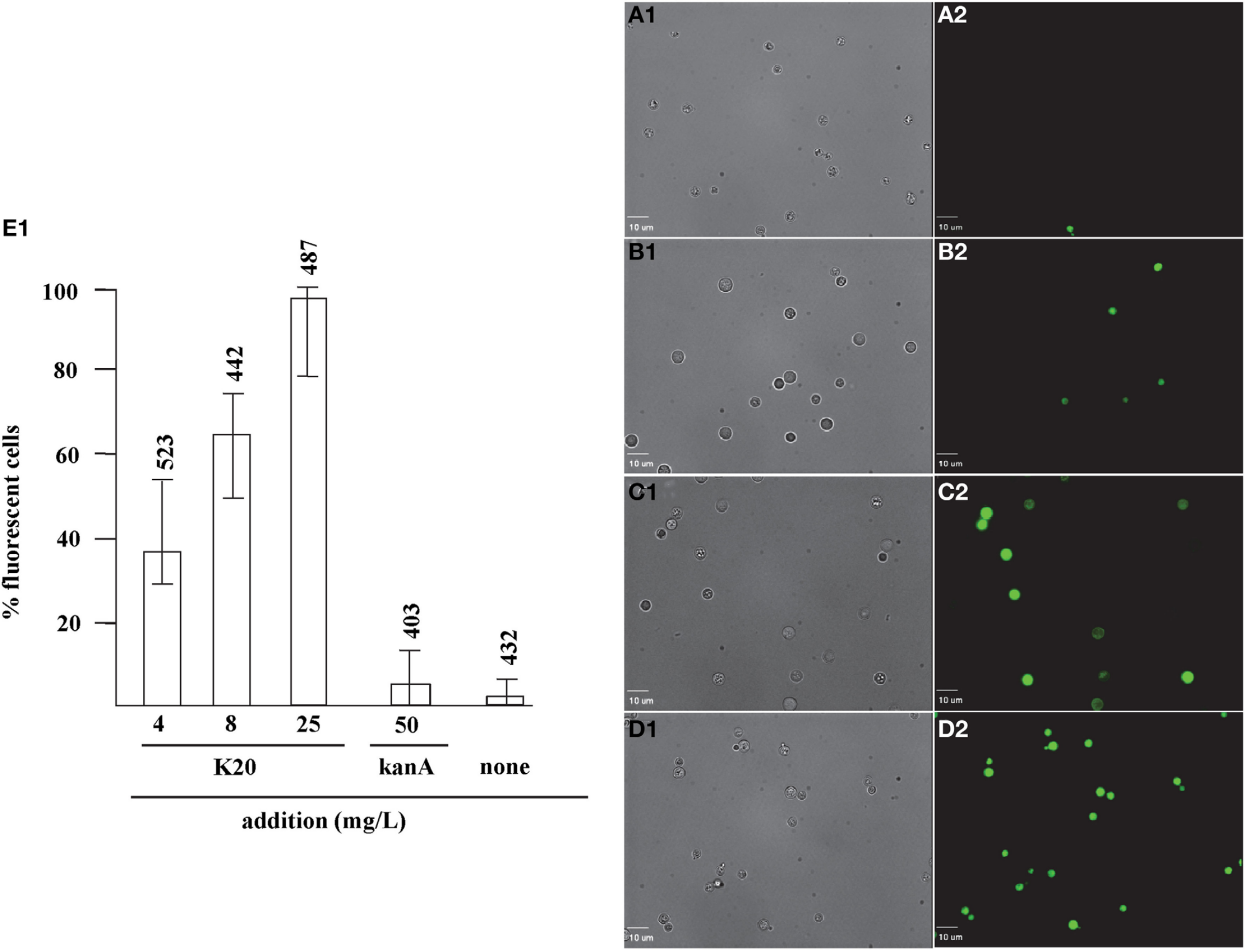
**Dose-dependent membrane perturbation effects of K20 on *C. neoformans* H99**. FITC dye uptake without **(A1,A2)** and with K20 (4 mg/L) **(B1,B2)**, (8 mg/L) **(C1,C2)**, and (25 mg/L) **(D1,D2)** exposure for 10 min. Bright-field images **(A1,B1,C1,D1)** are compared with fluorescence images **(A2,B2,C2,D2)**. Bar length is 10 μm. **(E1)** Shows dose-dependent effects of K20 on FITC dye uptake and effects of kanamycin A and no treatment Triton X-100^®^ (1%,vol/vol) gave 100% dye influx (data not shown). The error bars show SD from analyses of 10 separate microscopic image fields randomly selected from at least two separate experiments. Numbers above the range bars indicate the number of cells analyzed.

**Figure 7:**
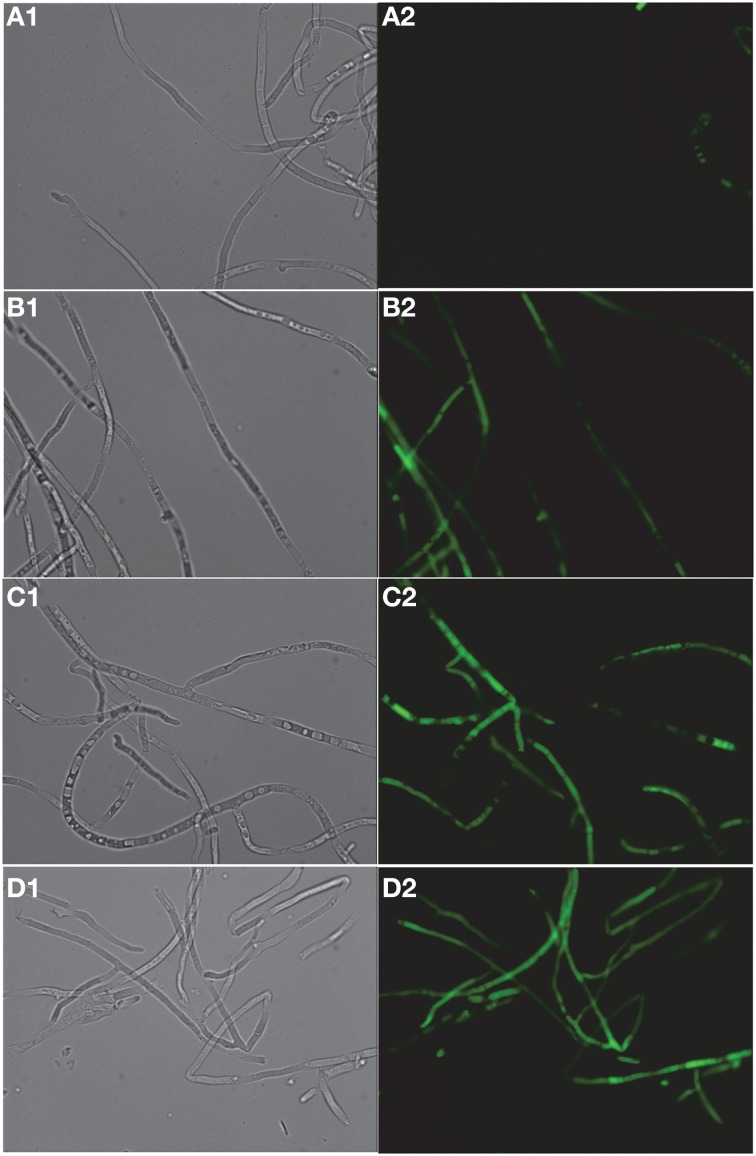
**Dose-dependent membrane perturbation effects of K20 on *F. graminearum***. FITC dye uptake without **(A1,A2)** and with K20 (7.8 mg/L) **(B1,B2)**, (15.6 mg/L) **(C1,C2)**, and (32 mg/L) **(D1,D2)** exposure for 10 min. Bright-field images **(A1,B1,C1,D1)** and fluorescence images **(A2,B2,C2,D2)**. Image a2 (no antimicrobial agent added) shows no fluorescent cells against a fluorescent background. Bar length is 10 μm. Triton X-100^®^ (1%, vol/vol) was assumed to give 100% dye influx (data not shown).

FITC dye was used to assess the membrane-perturbation effects of K20 on the plasma membrane of *C. neoformans* H99 and *F. graminearum*. FITC traverses cell surface membranes damaged or permeabilized by external agents and concentrates intracellularly to impart green fluorescence (Grilley et al., 1998; Mangoni et al., 2004). For *C. neoformans* H99, K20 at 8 and 25 mg/L caused FITC staining of 64 and 100% of the cells, respectively, and <5% when exposed to kanamycin A (50 mg/L) (Figure 6). Untreated cells were negligibly stained (<2%). With *F. graminearum* B4-A5 hyphae, quantitation of the number of FITC stained cells was difficult because of its multinucleated cell structure. Qualitatively however, 15.6 and 32 mg/L K20 were observed to cause a high degree of FITC cell staining compared to exposure to 50 mg/L kanamycin A that gave essentially no visible staining of hyphae (Figure 7).

### SUV calcein release

**Figure 8:**
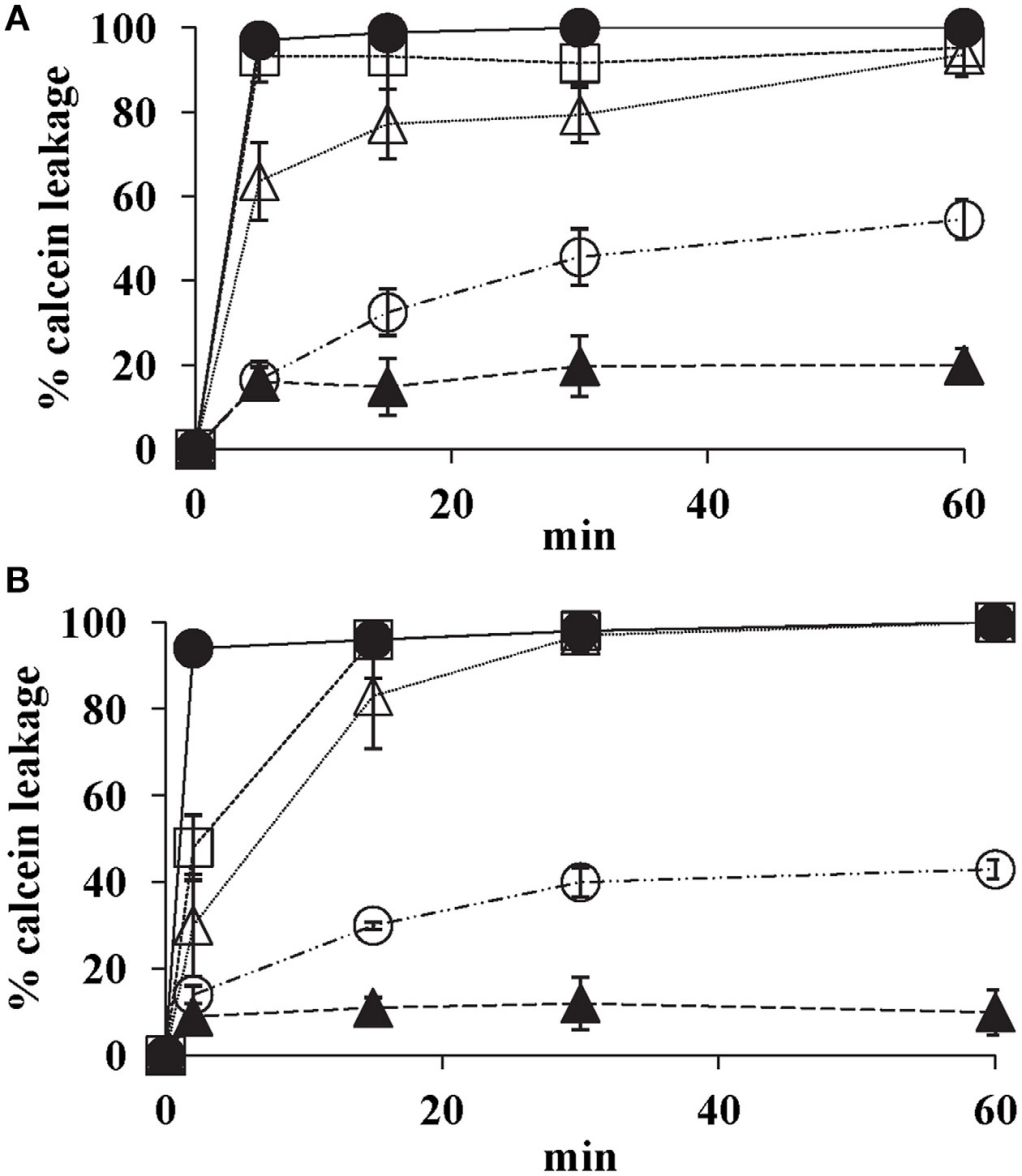
**Effect of K20 on calcein release from SUVs that mimic fungal plasma membranes**. Calcein released from SUVs made with PC/PE/PI/ergosterol (5:4:1:2) **(A)** and PC/ergosterol (7:3) **(B)** were exposed to K20 at 31.3 mg/L (open circles), 62.5 mg/L (open triangles), and 125 mg/L (open squares), kanamycin A at 62.5 mg/L (filled triangles), and Triton X-100^®^ (1% vol/vol) (filled circles). Data were compiled from three separate experiments. Standard deviation was used as the statistical parameter.

K20 showed dose- dependent release of calcein from model lipid bilayer membrane SUVs that mimic fungal plasma membranes. Within 15 min, K20 at 31.2 mg/L caused 30% calcein leakage from SUVs composed of PC, PE, PI, and ergosterol (5:4:1:2 by wt) and of PC and ergosterol (7:3 by wt) (Figure 8). At 62.5 mg/L, K20 caused 70–80% leakage from both types of SUVs within 15 min (Figure 8). SUVs without added K20 or treated with Triton X-100**®** showed <10 or 100% calcein leakage, respectively.

### Susceptibility of *S. cerevisiae* sphingolipid biosynthetic mutants

Fungal cell surface sphingolipids influence the inhibitory activities of several amphiphilic, membrane interacting antifungal compounds (Grilley et al., 1998; Stock et al., 2000; Thevissen et al., 2003, 2012; Sugimoto et al., 2004). Among these compounds is syringomycin E (Segre et al., 1989) which resembles K20 in size (<1500 daltons) and structural features of hydrophilic domains rich in hydroxyl and amino groups and hydrophobic domains composed of alkyl chains. Fungal mutants with aberrations in sphingolipid structure or composition have been used to reveal the roles of these lipids in antifungal mechanism of action (Grilley et al., 1998; Stock et al., 2000; Ferket et al., 2003; Thevissen et al., 2003, 2012). Therefore, syringomycin E-resistant *S. cerevisiae* mutant strains with lipid defects caused by single gene disruptions in specific sphingolipid biosynthetic genes were examined for susceptibility to K20. Strain W303-Δ*syr2* lacks the C4-hydroxyl group of the phytosphingosine backbone, strain W303-Δ*elo3* has defective sphingolipids with truncated very long fatty acyl chains, and W303-Δ*syr4* (*ipt1*) lacks the most complex and abundant yeast sphingolipid, mannosyl-diinositolphosphoryl-phytoceramide (MIP_2_C) (Grilley et al., 1998; Stock et al., 2000). MICs against strains W303-Δ*syr2* and W303-Δ*elo3* were 4–and 2-fold higher, respectively, compared to those for isogenic wild-type strain W303C and strain W303-Δ*syr4* (*ipt1*) (Table 2).

**Table 2:** **K20 susceptibilities of *Saccharomyces cerevisiae* sphingolipid biosynthesis mutants^a^**.

**Strain and genotype**	**MIC (mg/L)^b^**
W303C (*MATα ade2 his3 leu2 trp1 ura3*)	15.6
W303-Δ*syr2* (*MATα ade2 his3 leu2 trp1 ura3 syr2 (sur2)::URA3*)	62.5
W303-Δ*elo3 (MATα ade2 his3 leu2 trp1 ura3 elo2::HIS3)*	31.3
W303-Δ*syr4 (ipt1)(MATα ade2 his3 leu2 trp1 ura3 syr4 (ipt1)::URA3*	15.6

a *Values are average of three assay determinations*.

b *Microbroth dilution assays were conducted in RPMI 1640 medium*.

### Effect on preventive murine lung infectivity of *C. neoformans* H99

**Figure 9:**
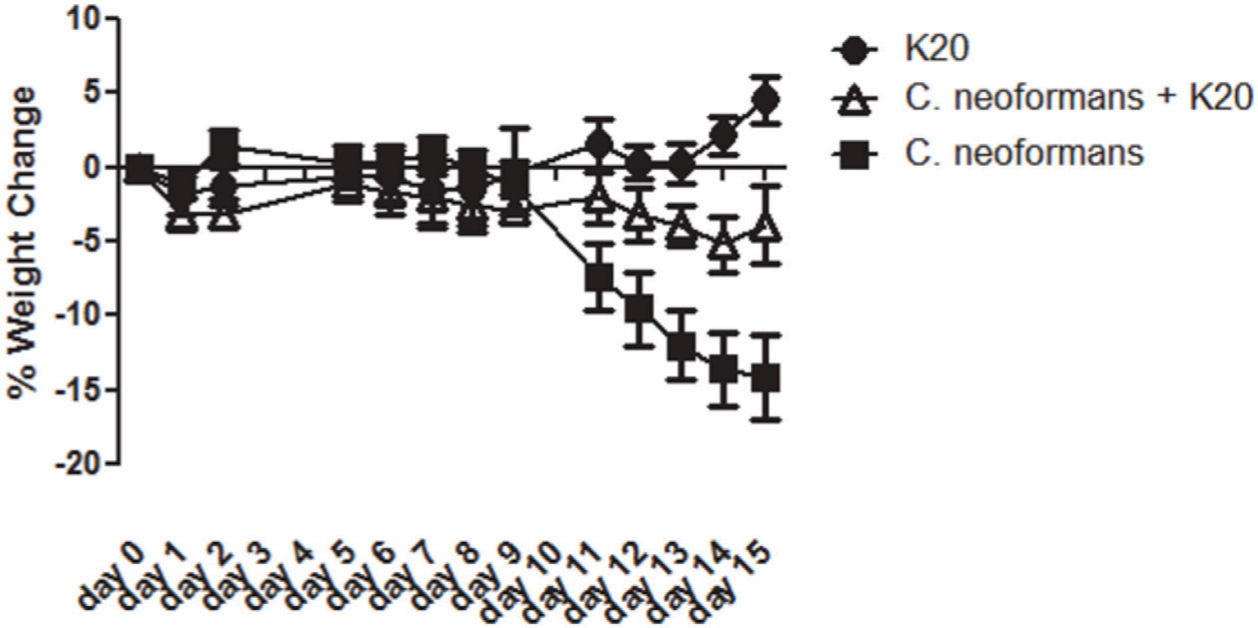
**Mean percent body weight change in groups of mice receiving treatments of K20**. Treatments with K20 were one dose/animal of 100 μL of 200 mg/L K20 (filled circles), infected with one dose/animal of 100 μL of *C. neoformans* H99 (5 × 10^3^ cells/mL) mixed with K20 (200 mg/L) (open triangles), and infected with 1 dose/animal of 100 μL *C. neoformans* H99 (5 × 10^3^ cells/mL) only (filled squares). Data were statistically analyzed and *P*-values determined by One-Way ANOVA methods.

**Figure 10:**
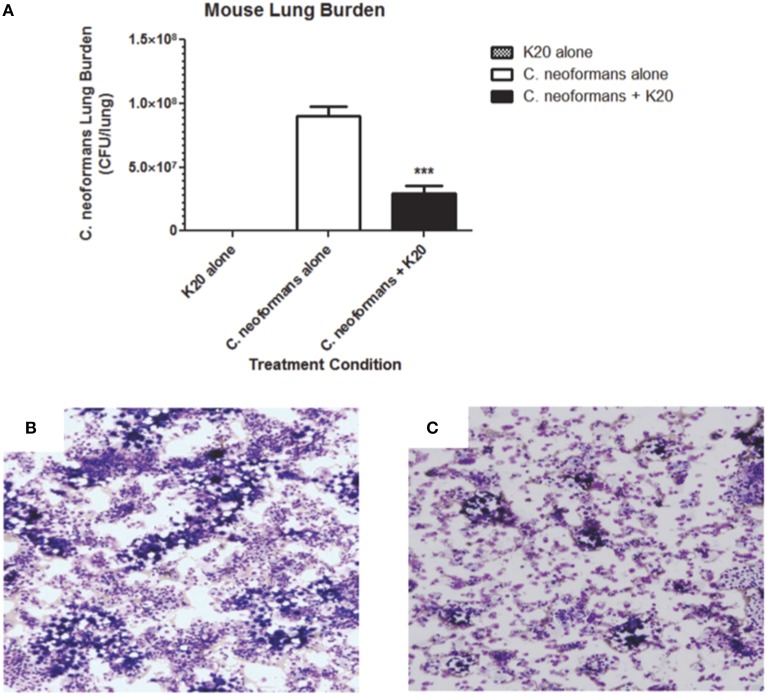
**Effect of K20 on *C. neoformans* H99 infectivity in a RAG^−/−^ mouse model. (A)** Mouse lung fungal burden at day 15 after infection with *C. neoformans* H99 cells mixed (black bar) or not mixed (white bar) with K20. Fungal burdens were assessed by plating lung homogenate suspensions for CFU determinations. Control treatment with K20 and no cells (gray bar) showed no fungal burden. ^***^ Indicates a *p*-value < 0.001 determined by One-Way ANOVA. Light microscopic images of lung homogenates were prepared from *C. neoformans* H99 infected mice with no K20 **(B)** and with K20 **(C)** exposure. Methanol-fixed homogenates on glass slides were stained with Diff-Quik™. Yeast cells were visible as large purple colored cells surrounded by an opaque halo.

RAG^−/−^ mice treated intratracheally with a mixture of K20 and *C. neoformans* H99 cells maintained their body weights over a 15-day infection time course. In contrast, mice treated with *C. neoformans* H99 cells only showed weight losses starting at day 10 post-infection (Figure 9). Lung fungal burdens of infected mice were 4-fold (*p* ≤ 0.01) lower with K20 treatment in comparison to untreated infected mice (Figure 10A). Stained images of lung homogenates showed qualitatively decreased fungal burdens in mice infected with the mixture as compared to mice receiving cells only (Figures 10B,C).

## Discussion

Unlike the difficult and complex synthesis of FG08, the synthesis of K20 is simple and efficient. K20 synthesis involves direct modification of kanamycin A and fewer synthetic steps. Readily available reagents and large stockpiles of kanamycin A (starting material) for its synthesis enhance the prospects for its scalable production (laboratory scale of ~300 g per batch) and use. For both FG08 and K20, the antibacterial capabilities of the corresponding kanamycin (kanamycin B for FG08 and kanamycin A for K20) are simultaneously diminished with C8 alkyl chain attachment. Thus, a “switch“ occurs from bacterial to fungal growth inhibition corresponding to non-alkylated and C8 alkylated kanamycin derivatives, respectively (Table 1). Lacking antibacterial activities, K20 is not expected to promote bacterial resistance with environmental or therapeutic use—a major concern with the use of its parent kanamycin A (Fisher et al., 2012).

Among the fungi species examined, *Aspergillus* species were least susceptible to inhibition by K20 (Table 1). In contrast, all yeasts tested were growth inhibited by K20 with *C. neoformans* strains showing relatively high degrees of susceptibility. Fusarium species, *F. gramineaum* and *F. oxysporum* were also highly susceptible showing that K20 has broad spectrum antifungal inhibitory capabilities that include filamentous fungi as well as yeast. The generally higher MICs observed with K20 as opposed to the lower MICs for azoles (Table 1) reflect the different modes of action of these antifungal agents. The antifungal MICs observed with K20 are similar to those achieved with recently reported amphiphilic terephalamide–bisurea (Fukushima et al., 2013) and lipopeptides (Makovitzki et al., 2008; Vallon-Eberhard et al., 2008) that are antifungal when they assume high-aspect ratio supramolecular assemblies that interact with target membranes. The comparatively higher antifungal MICs of these amphiphiles and possibly K20 are a consequence of polymeric assembly formation that precedes pore formation in membranes. Consistent with its observed anti-cryptococcal activity, K20 significantly reduced the infectivity of *C. neoformans* H99 in mice when administered intratracheally together with the pathogen showing that even a single initial treatment of K20 made a significant difference (*p* = 0.01) in the propagation of the fungal pathogen. Finally, it is noted that K20 inhibited well-known azole resistant strains *C. albicans* ATCC 64124, *C. albicans* B-311, and *C. tropicalis* 95-41 with MICs that were lower than those of itraconazole and fluconazole (Table 1). Therefore, it appears that K20 is less subject to the azole resistance mechanisms of *C. albicans* ATCC 64124 and B-311 and *C. tropicalis* 95-41.

With an octanesulfonyl chain at the *O*-6″ position, K20 is an amphiphilic compound which in turn suggests interaction with target cell membranes. Kanamycin A, K20′s parent compound has an anti-bacterial mechanism of action that causes protein translation misreading. K20′s rapid killing observed in the time-kill curve studies (~10^3^-fold CFU decrease in 4 h at 8 mg/L) (Figure 3) suggests direct membrane action as the basis for growth inhibition rather than indirect and slower effects elicited by protein translational misreading or other indirect effects that require cellular processing. Two approaches were used to assess membrane-perturbation effects of K20. Membrane-impermeable FITC uptake studies with cells and calcein leakage studies conducted with model lipid bilayer SUVs suggest that K20′s growth inhibitory effect is due to direct and rapid effects on plasma membrane permeability. These membrane perturbing effects resemble those previously observed with K20′s predecessor, FG08 (Shrestha et al., 2013). Therefore, the conversion of kanamycin A to K20 not only alters the group of organisms it inhibits but also the mode of action.

Altered K20 susceptibilities of yeast mutants with defective sphingolipids further support interaction with the fungal plasma membrane. Yeast sphingolipids are mainly located in plasma membranes, and they possess fungal-specific structural features that allow high densities of hydrogen and ionic bonding sites for potential interaction with K20 (Stock et al., 2000). *S. cerevisiae* strain W303-Δ*syr2* which lacks the sphinganine backbone C4 hydroxyl group (Grilley et al., 1998) and strain W303-Δ*elo3* with truncated fatty acid tails were 4 and 2-fold less sensitive to K20 compared to the isogenic wild-type strain W303C (Table 2). These observations indicate a role for sphinganine C4 hydroxylated sphingolipids in promoting K20 action on the yeast plasma membrane. Yeast sphingolipids differ structurally from the mammalian sphingolipids and also bacterial membrane lipids (Lester and Dickson, 1993). The former have C4-hydroxylated sphinganine backbones and many have inositolphosphate-containing head groups—features not found in mammalian or bacterial cell lipids These structural differences may account for K20′s preferential targeting of yeast and other fungi vs. mammalian and bacterial cells. An absolute requirement for sphingolipids in K20 action, however, is unlikely. SUVs used in this study lacked sphingolipids and were still permeabilized by K20 (Figure 8). It is more likely that combinations of lipids and other membrane components that confer favorable interaction sites (such as, but not exclusively, sphingolipids) are responsible for K20 binding and action. Similar sphingolipid-promoting, membrane pore forming modes of action may be speculated for antifungal syringomycin E (Grilley et al., 1998; Stock et al., 2000) and the plant defensin DmAMP1 (Thevissen et al., 2000; Im et al., 2003). In contrast, K20′s rapid permeability effect on non-sphingolipid containing SUVs indicates that its mechanism of action differs from that of antifungal plant defensin RsAPF2 (Thevissen et al., 2012). RsAPF2 increases fungal membrane permeability by generating reactive oxygen species following binding to the sphingolipid glucosylceramide (Aerts et al., 2007; Thevissen et al., 2012).

In conclusion, a novel aminoglycoside analog of kanamycin A, K20, with an octanesulfonyl chain as a major structural feature, is a broad-spectrum antifungal that targets fungal plasma membranes. K20 is not hemolytic, showed low mammalian cell toxicities, and it reduced cryptococcal lung infectivity in a mouse model. Because of these features, K20 is suggested as a lead compound for a novel class of therapeutic antifungals as well as crop protectants in agriculture.

### Conflict of interest statement

The authors declare that the research was conducted in the absence of any commercial or financial relationships that could be construed as a potential conflict of interest.
