# Supplementary material for: Antifungal amphiphilic aminoglycoside K20: bioactivities and mechanism of action
Source: Front Microbiol. 2014 Dec 5;5:671. doi: 10.3389/fmicb.2014.00671 (PMC4257101; doi:10.3389/fmicb.2014.00671)
Supplement: Supplementary file 1 [file DataSheet1.DOCX]

Supplemental Material

K20 STRUCTURE DETERMINATION

**METHODS**

K20 was characterized by ^1^H NMR, ^13^C NMR and mass spectrometry. Correlation Spectroscopy (COSY) and edited Heteronuclear Single Quantum Correlation (HSQC) were used for H-H and H-C correlation respectively. The edited COSY provides information for the identification of protons (H-1ʺ, H-2ʺ, H-3ʺ, H-4ʺ, H-5ʺ and H-6ʺ) on ring III of K20. The CH_2_ groups are coded in red; and CH and CH_3_ are coded in green in HSQC spectrum.

**RESULTS**

**^1^H and ^13^C NMR spectroscopy.** ^1^H NMR spectra (Fig. 1A-D) showed only two anomeric protons indicating a high level of purity. The extent of purity is supported by High Resolution Mass Spectrometry (HRMS). Normal (Fig. 1E) and expanded (Fig. 1F,G) ^13^C NMR spectra reveal two sets of quartets (110.5-1232.1ppm; 162 ppm) belonging to triflate which is a counter ion during ^13^C NMR analysis. Due to the lack of a chromophore, it was not possible to use high performance liquid chromatography for purity analyses.


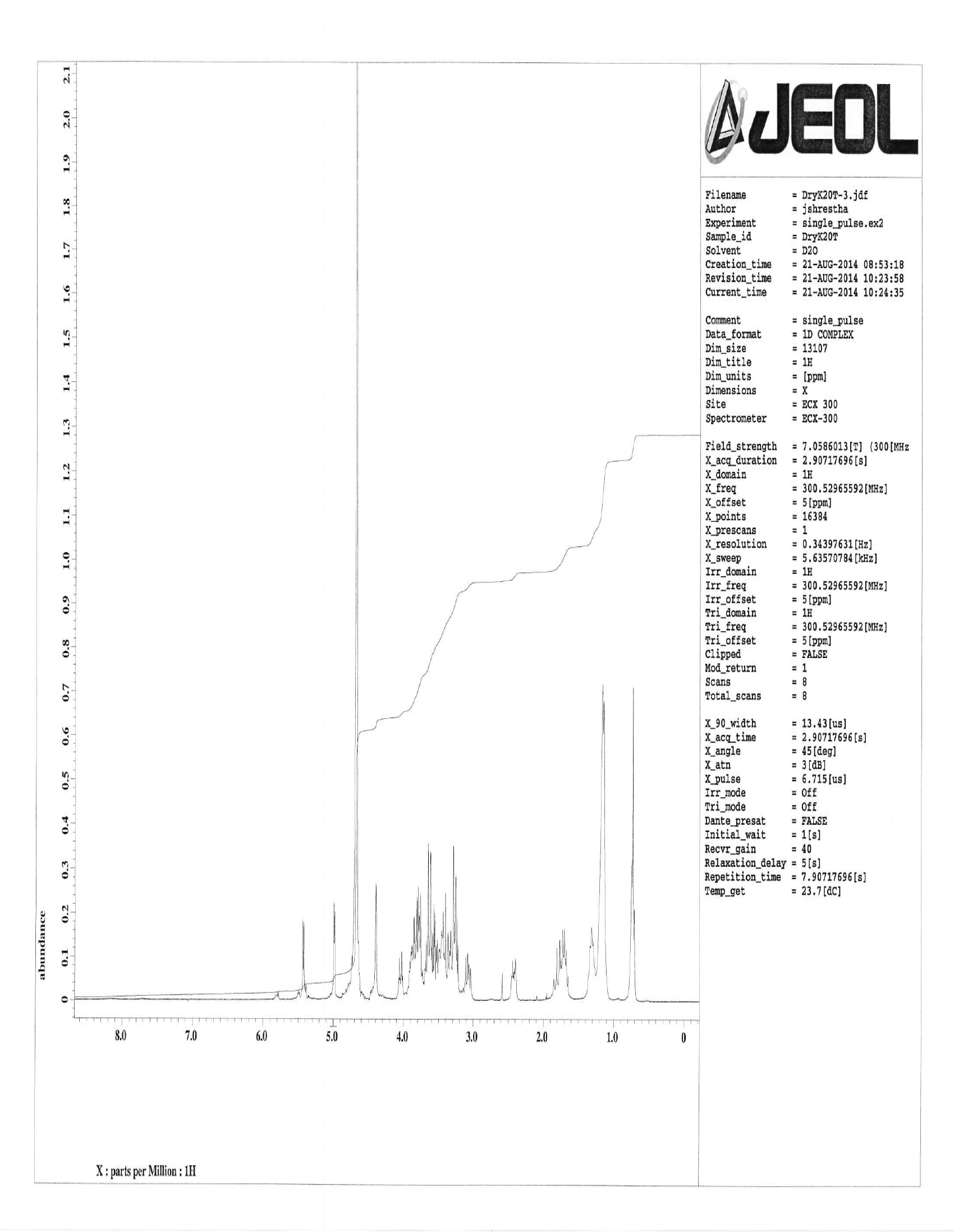


A

**
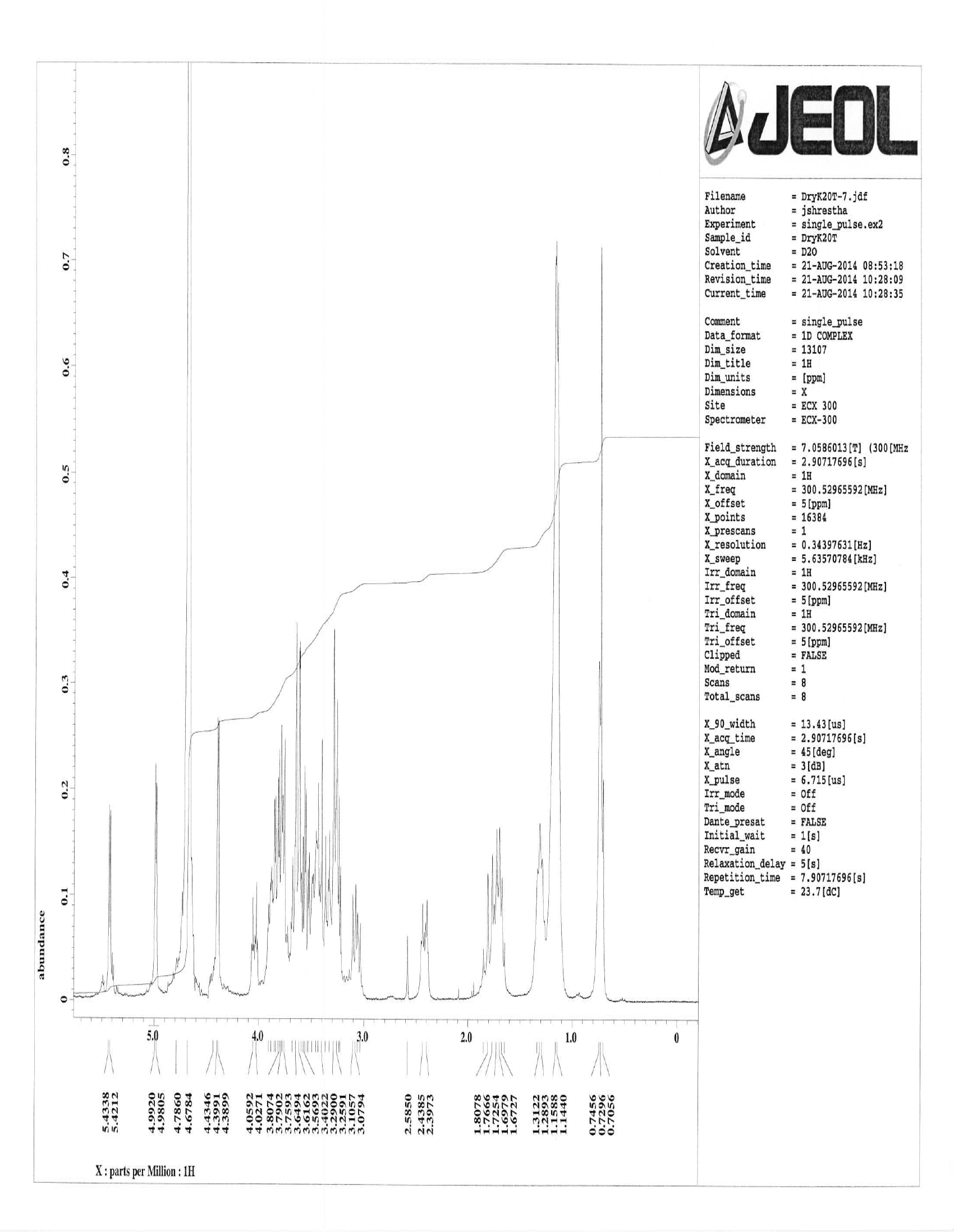
**

B

**
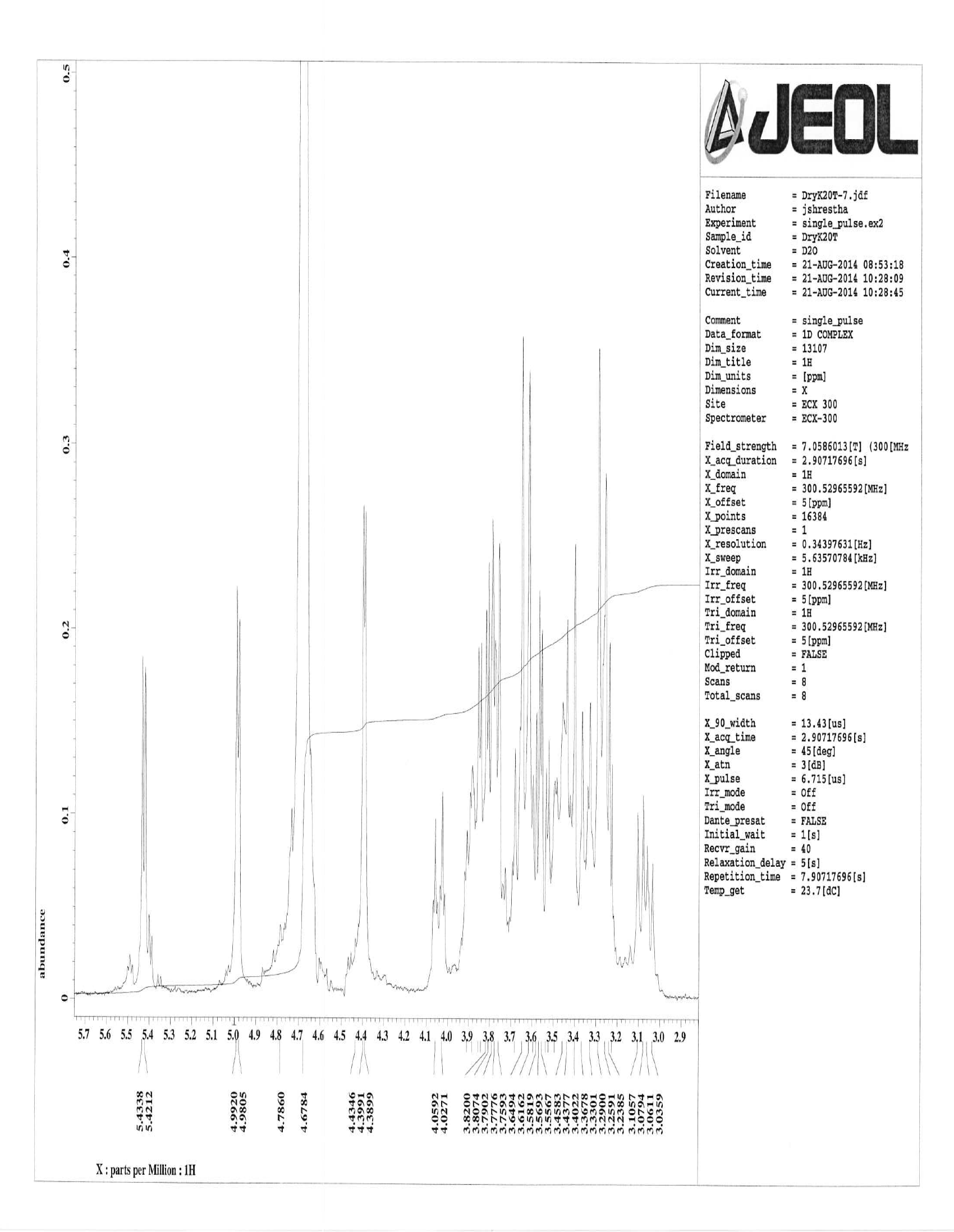
**

C

**
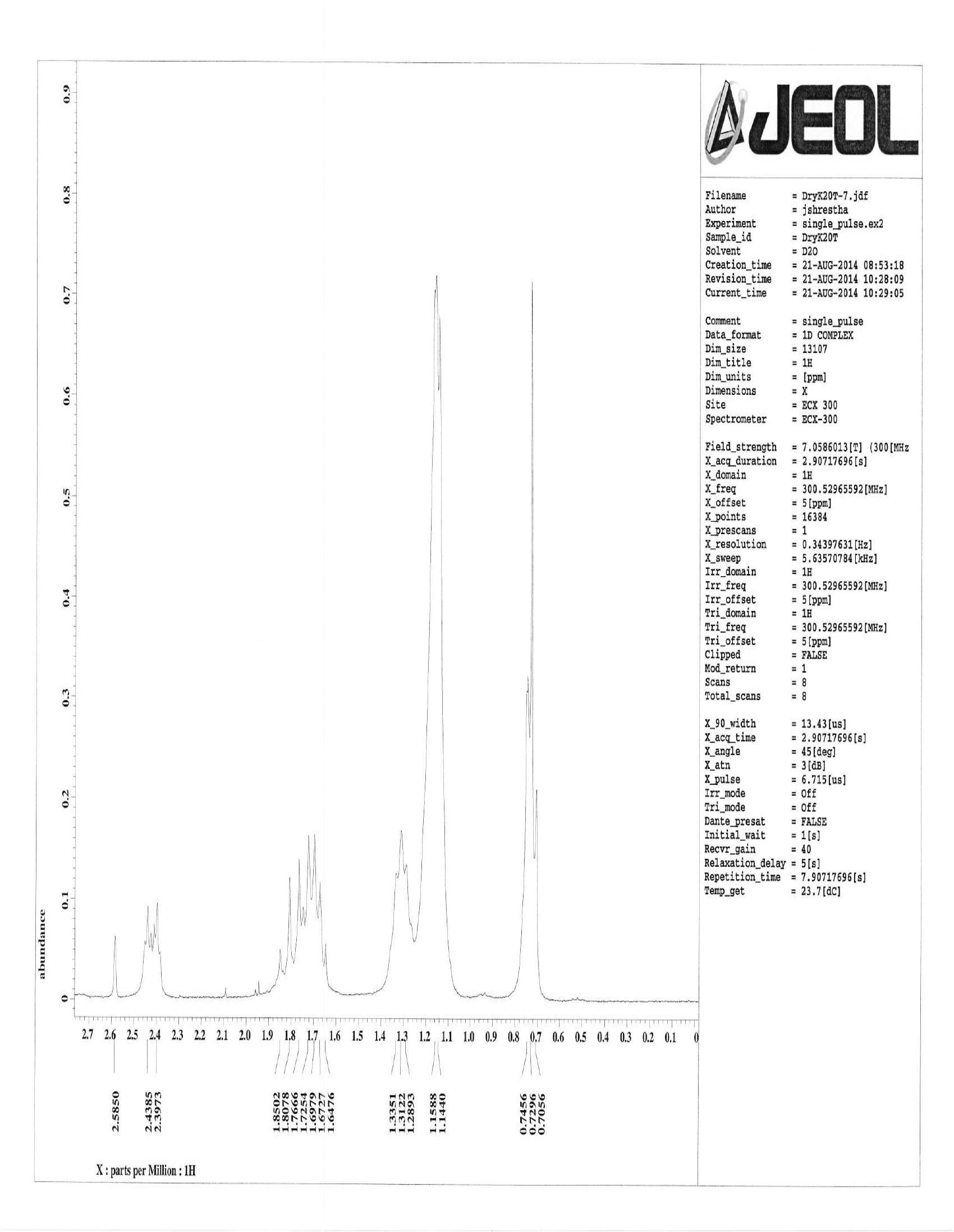
**

D

**
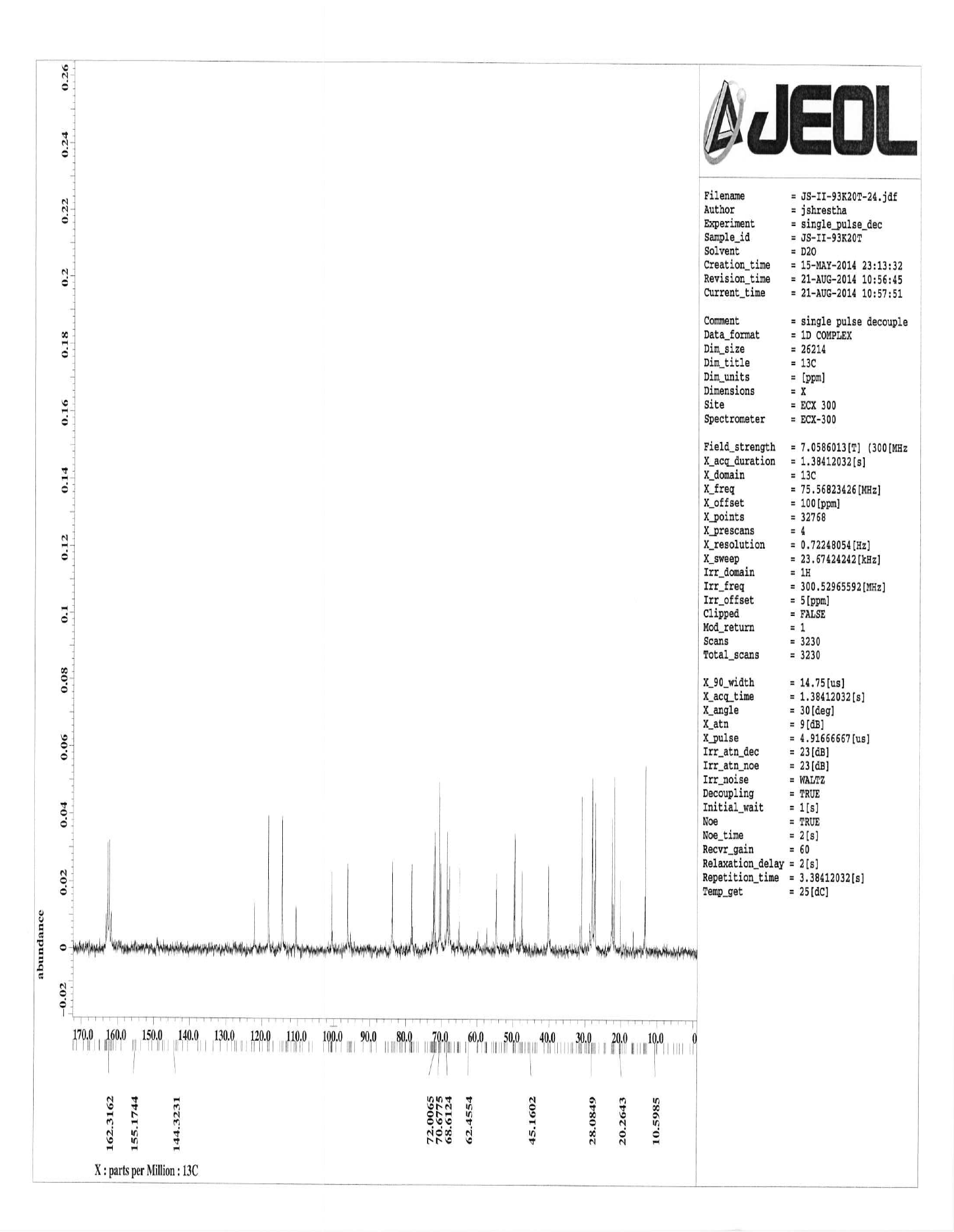

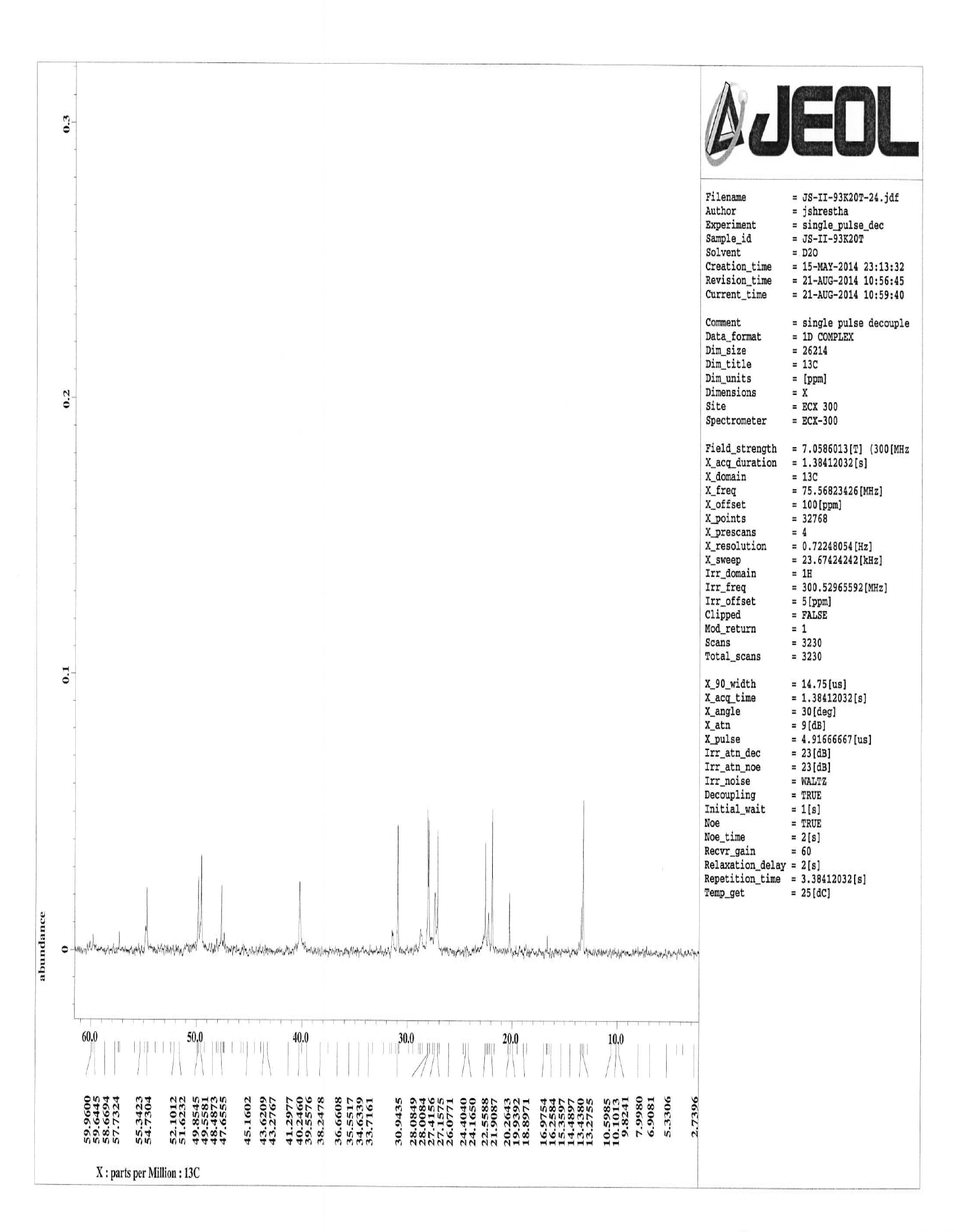
**

E

F

**
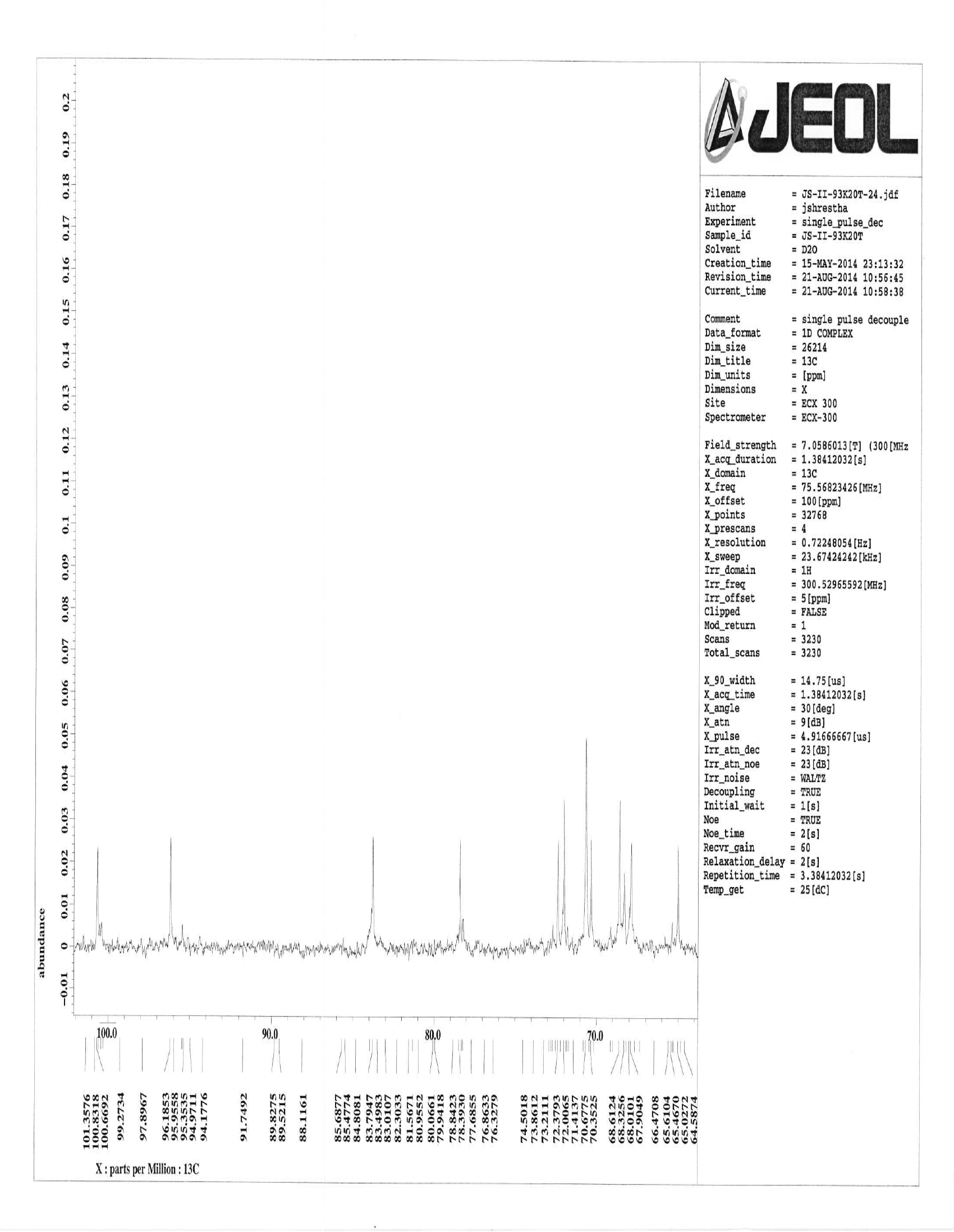
**

G

**Figure 1.** ^1^H (panels **A–D**) and ^13^C NMR (panels **E-G**) spectroscopic data for K20. High Resolution Mass Spectrometry (HRMS) data are shown in panels **B-D** and expanded ^13^C NMR spectra are shown in panels **F** and **G**. Spectroscopy data analysis: **(A)** ^1^H NMR (300 MHz, D_2_O) (Triflate salt) δ 5.43 (d, *J* = 3.8 Hz, 1H), 4.99 (d, *J* = 3.5 Hz, 1H), 4.39 (d, *J* = 2.8 Hz, 2H), 4.04 (ddd, *J* = 10.0 Hz, 3.3 Hz, 3.3 Hz, 1H), 3.2 – 3.9 (m, 15H), 3.10 (dd, *J* = 13.4 Hz, 9.5 Hz, 1H), 2.40 – 2.43 (ddd, *J* = 12.4 Hz, 4.0 Hz, 4.0 Hz, 1H), 1.6 – 1.9 (m, 3H), 1.65– 1.85 (m, 2H), 1.16 – 1.14 (m, 8H), 0.71-0.75 (t, *J* = 4.8 Hz, 3H).  **(B)** ^13^C NMR (75 MHz, D_2_O) (Triflate salt) δ 100.8, 96.2, 83.8, 78.4, 72.4, 72.0, 70.7, 70.4, 68.6, 68.3, 67.9, 65.0, 54.7, 49.9, 49.6, 47.7, 40.2, 31.0, 28.0 (2C), 27.4, 27.2, 22.6, 22.0, 20.3, 13.3.

Figure 2. COSY spectrum of K20.

Figure 3. HSQC spectrum of K20

**Mass spectral analysis**. The ESI/APCI calculation for C_26_H_53_N_4_O_13_^+^ ([MH]^+^) was m/z 661.3324. The measured m/z was 661.3319.
